# Electrochemical CO_2_ Reduction Using Copper–Zinc and Copper–Bismuth Catalysts: Mechanistic Insights and Design Perspectives

**DOI:** 10.1002/tcr.202500131

**Published:** 2025-10-06

**Authors:** Elías Mardones‐Herrera, Ilaria Gamba, Mauricio Isaacs, Gonzalo García

**Affiliations:** ^1^ Departamento de Química Instituto Universitario de Materiales y Nanotecnología Universidad de La Laguna P.O. Box 456 Santa Cruz de Tenerife 38200 Spain; ^2^ Facultad de Química Pontificia Universidad Católica de Chile Vicuña Mackenna 4860 Macul Santiago 7820436 Chile; ^3^ Millennium Institute on Green Ammonia as Energy Vector‐MIGA Pontificia Universidad Católica de Chile Santiago 7820436 Chile

**Keywords:** catalysis, Cu–Bi catalysts, Cu–Zn catalysts, electrochemical reduction of CO_2_, electrocatalysis

## Abstract

The electrochemical reduction of CO_2_ (ECO_2_RR) has emerged as a promising route for converting CO_2_ into value‐added chemicals and fuels using renewable electricity. Among transition metals, copper uniquely enables the formation of C_1_ and C_2_ products, but it suffers from poor selectivity and stability in its monometallic form. This review explores recent advances in Cu–Zn and Cu–Bi bimetallic systems, emphasizing how their structural, electronic, and interfacial characteristics influence ECO_2_RR pathways and product distribution. Mechanistic insights into active site behavior, alloying effects, and the role of surface facets are provided. Limitations such as catalyst degradation and scalability are critically discussed, including a comparing performance across different electrochemical cell configurations. Finally, design guidelines and future research directions are proposed to enhance Cu‐based bimetallic catalysts’ selectivity, stability, and scalability for ECO_2_RR.

## Graphical abstract

This review explores tandem copper‐based catalysts combining Cu–Bi and Cu–Zn interfaces for CO_2_ electroreduction. Synergistic effects guide product selectivity: Cu–Bi promotes formate formation, while Cu–Zn enhances *CO generation for C_2_ product pathways.

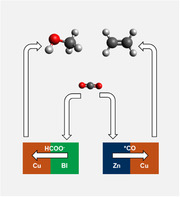

## 1 Introduction

Burning fossil fuels for energy releases too much carbon dioxide (CO_2_) into the air, raising the atmospheric CO_2_ concentration to 427 ppm in 2025.^[^ ^1^ ^]^ This increase has contributed to global warming and the alarming retreat of glaciers.^[^ ^2^, ^3^ ^]^ The higher amount of CO_2_ in the atmosphere absorbs the infrared radiation reemitted by the Earth, creating a thermal insulation effect leading to the greenhouse effect and a rise in the atmospheric temperature.^[^ ^2^ ^]^

The growing threat of global warming and environmental degradation underscores the need to implement clean and efficient strategies to reduce the global carbon footprint. Among these, carbon capture and storage is considered one of the most effective approaches to lowering CO_2_ emissions.^[^ ^4^, ^5^ ^]^ In this context, CO_2_ activation and hydrogenation to produce hydrocarbons and oxygenated compounds—such as alcohols—represent a promising pathway to recycle, store, and convert emissions into value‐added products.^[^ ^6^, ^7^, ^8^, ^9^ ^]^

This review provides a focused and comprehensive overview of recent progress in the electrochemical reduction of CO_2_ using copper‐based bimetallic systems, particularly Cu–Zn and Cu–Bi catalysts. We examine how these secondary metals’ structural, electronic, and surface modifications influence catalytic performance, reaction pathways, and product selectivity. The review primarily covers experimental and theoretical studies published between 2019 and 2024, emphasizing structure–activity relationships, mechanistic insights, and the impact of cell configuration in gas‐ and liquid‐phase electrolysis.

The electrocatalytic reduction of CO_2_ (ECO_2_RR) has gained significant attention as a sustainable, low‐temperature strategy to convert CO_2_ into valuable fuels and chemicals.^[^ ^10^ ^]^ Integrating carbon into circular processes helps mitigate the environmental impact of anthropogenic emissions.^[^ ^11^ ^]^ Beyond its sustainability, ECO_2_RR enables the selective formation of valuable products such as methanol, ethylene, and other hydrocarbons, depending on the catalyst used.^[^ ^12^, ^13^ ^]^

One common strategy involves forming oxide films on metallic copper surfaces to prepare structured electrodes with improved activity.^[^ ^14^, ^15^ ^]^ Copper‐based catalysts are especially promising for enhancing selectivity toward oxygenated products, particularly methanol.^[^ ^16^ ^]^ The COOH intermediate has been identified as a key species in the methanol formation pathway.^[^ ^17^ ^]^ While absorbed carbon monoxide (CO) is a significant intermediate in the reaction, it plays a central role in the formation of both C_1_ products (i.e., methane) and C_2_ products (i.e., ethylene and ethanol).^[^ ^18^ ^]^ Hydroxyethylidene (OCCOH) formation is also critical for producing multicarbon products, as it arises from CO–CO coupling. Copper's unique electronic and structural properties make it a particularly promising catalyst for addressing the selectivity challenges of ECO_2_RR.^[^ ^19^ ^]^

However, the rapid deactivation of copper electrodes during operation suggests that surface oxide films are either reduced or transformed into other active species under reaction conditions.^[^ ^20^ ^]^ Copper oxide (CuO) has attracted particular interest due to its ability to promote the formation of oxygenated products such as ethanol and ethylene. Ethylene generation has been linked to the dimerization of CO, which forms hydroxycarbonyl (OCCOH) intermediates.^[^ ^21^ ^]^ These intermediates can adopt different configurations depending on their interaction with the catalyst surface, influencing the product pathway. Competing CO dimerization routes may also lead to alternative intermediates, which guide the reaction toward either ethylene or ethanol.^[^ ^22^, ^23^ ^]^

Although copper‐based catalysts have shown promising results, achieving high efficiency in CO_2_ reduction remains a challenge. To address this, recent studies have explored bimetallic Cu‐based systems, where the addition of a secondary metal such as zinc (Zn) or bismuth (Bi) modifies the electronic and geometric properties of the catalyst, enhancing both activity and selectivity.^[^ ^24^, ^25^ ^]^ This review highlights recent developments involving Cu–Zn and Cu–Bi catalysts, illustrating how these combinations outperform monometallic copper in ECO_2_RR applications.

## 2 Cu–Zn and Cu–Bi Catalysts for ECO_2_RR

Incorporating Zn and Bi into Cu‐based catalysts significantly alters their electronic and structural properties, enhancing selectivity for CO_2_ reduction. Zn promotes CO evolution, favoring the formation of hydrocarbons such as ethylene (C_2_H_4_),^[^ ^26^, ^27^ ^]^ while Bi stabilizes oxygenated intermediates, thereby facilitating the production of methanol (CH_3_OH) and formate (HCOO^−^).^[^ ^28^, ^29^ ^]^ The following sections discuss how Zn and Bi influence catalyst structure and activity and compare the performance of these bimetallic systems with that of monometallic Cu.

### 2.1 Cu–Zn Catalysts: Enhancing CO and C_2_ Product Formation

**Figure 1:**
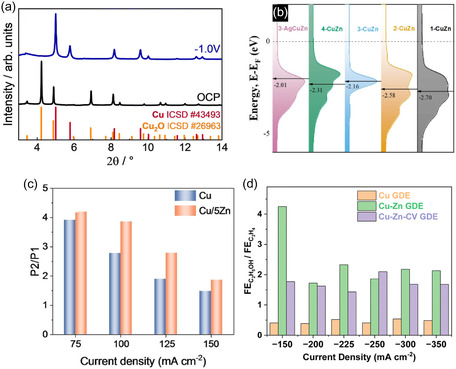
a) X‐ray diffraction patterns of Cu and Cu–Zn catalysts. Reproduced from ref. [31] published under a Creative Commons Attribution License (CC BY 3.0). b) PDOS for Cu in different Cu–Zn compositions and Ag‐modified Cu–Zn. Reproduced from ref. [32] published under a Creative Commons Attribution License (CC BY 4.0). c) FE for the Cu–Zn catalyst's ethanol, methane, and carbon monoxide (CO) during electrochemical CO_2_ reduction at different current densities. Adapted with permission.^[^ ^33^ ^]^ Copyright 2023, John Wiley & Sons under the STM Permissions Guidelines. d) Under high‐current‐density conditions (1.1 A cm^−2^), the Cu/Zn catalyst exhibited a maximum FE of 50.7% toward C_2_ alcohols, including ethanol. Reproduced from ref. [34] published under a Creative Commons Attribution License (CC BY 3.0).

Incorporating Zn into the Cu lattice modifies the catalyst structurally and electronically, directly influencing its performance in CO_2_ electroreduction. Although Zn and Cu have similar atomic radii, the presence of Zn can induce lattice strain, which alters the adsorption behavior of CO on the surface. These structural and electronic shifts enhance C_2_ products such as ethylene and ethanol formation.^[^ ^30^ ^]^ **Figure** **1**A illustrates the structural evolution of Cu‐based catalysts under open circuit potential (OCP) and during CO_2_ electroreduction at−1.0 V vs RHE. Cu_2_O diffraction peaks are initially observed under OCP but gradually disappear as the applied potential becomes more negative, indicating the reduction of the cuprous phase to metallic Cu. The observed peak shifts and broadening suggest lattice expansion and the presence of electrochemically induced strain, potentially caused by adsorbed species (such as CO or OH^−^) or the formation of transient alloy phases.^[^ ^31^ ^]^ The influence of Zn on the electronic structure is equally significant. As shown in Figure 1B, the projected density of states (PDOS) for Cu varies across different Cu–Zn compositions, including a silver‐modified variant. The catalysts—designated 1‐CuZn, 2‐CuZn, 3‐CuZn, and 4‐CuZn—contain 5%, 15%, 30%, and 50% Zn, respectively, while 3‐AgCuZn refers to the 3‐CuZn sample coated with silver. Increasing the Zn content shifts the d‐band center toward less negative values (from–2.70 eV in 1‐CuZn to–2.01 eV in 3‐AgCuZn), thereby weakening the binding energy of CO and facilitating its desorption. This shift weakens the interaction with CO, facilitating its desorption and thereby promoting the formation of C_2_ products during CO_2_ electroreduction.^[^ ^32^ ^]^ Zinc undermines CO adsorption and may function as a spatial and electronic modulator within the catalyst matrix. This dual role can be strategically utilized in the design of catalysts where Zn‐enriched domains promote CO dimerization, while metallic Cu regions provide effective CO_2_ activation. The resulting spatial synergy may account for the superior selectivity toward C_2_ alcohols observed with moderate Zn loadings compared to pure Cu under high‐current‐density conditions. These findings support the interpretation of Zn as an active cocatalytic component that influences both reactivity and selectivity in Cu‐based systems.

Experimental observations further reinforce these findings. As shown in Figure 1C, the ratio between higher‐order and lower‐order reduction products—P_2_/P_1_—was evaluated for Cu and Cu/5Zn catalysts under various current densities. In this context, P_2_ corresponds to multicarbon products such as ethanol, while P_1_ represents single‐carbon products like CO and formate. The significant increase in the P_2_/P_1_ ratio for the Cu/5Zn catalyst under high‐current‐density operation indicates a shift in product selectivity toward more complex multicarbon alcohols. Rather than reporting individual Faradaic efficiencies (FEs), this figure highlights how Zn incorporation alters the reaction pathway, favoring the formation of C_2_ products. These results underscore the critical role of Zn in promoting C—C coupling and enhancing the production of higher‐value products, demonstrating its potential in the rational design of scalable Cu‐based electrocatalysts for CO_2_ reduction.^[^ ^33^ ^]^ Based on these insights, the role of Zn can be interpreted as extending beyond that of a simple electronic dopant. Its capacity to modulate the electronic density of states of Cu implies a dual function: facilitating carbon monoxide dimerization while concurrently suppressing competing reactions such as the hydrogen evolution reaction (HER). This combined behavior is advantageous for rationally developing efficient electrocatalysts under industrially relevant conditions.

The performance of Cu–Zn catalysts strongly depends on the Zn content. Studies indicate that concentrations between 10% and 30% favor ethanol production, while higher Zn levels tend to increase CO evolution and reduce selectivity toward more complex C_2_ products.^[^ ^34^ ^]^ This trend is attributed to the promotion of CO formation at elevated Zn contents, which limits the further conversion of intermediates into multicarbon compounds.^[^ ^35^ ^]^

A Cu/Zn alloy with a 95:5 molar ratio (similar to the 1‐CuZn sample) achieved an FE of 50.7% for C_2_ alcohols during CO_2_ electrolysis. In such systems, low Zn concentrations promote CO adsorption at active Cu sites and facilitate hydrogen transfer from Zn to Cu. This synergistic interaction enhances C—C coupling, favoring the formation of multicarbon products such as ethanol and n‐propanol. In contrast, higher Zn loadings tend to promote the HER and increase ethylene production—both of which are less effective pathways for generating C_2_ alcohols,^[^ ^33^, ^34^ ^]^ Additionally, applying–1.05 V versus RHE to a Cu_4_Zn catalyst yielded a maximum FE of 29.1% for ethanol, confirming that moderate Zn content supports selective C_2_ formation by improving CO adsorption, conversion, and multicarbon coupling efficiency.^[^ ^34^ ^]^

**Table** **1** summarizes the FE (reaching up to ≈50%) achieved for producing C_2_ alcohols—particularly ethanol and methanol—using Cu–Zn catalysts. The most effective compositions typically contain around 5 mol percent Zn (Cu: Zn = 95:5), consistently demonstrating high selectivity toward multicarbon products at moderate to high‐current densities (≈100 mA cm^−2^). Catalysts with higher Zn content (10–30 mol percent) also produce appreciable amounts of ethylene and methanol, although with slightly lower efficiencies. All results were obtained under flow‐cell conditions, which enhance CO_2_ mass transport and enable higher current densities compared to conventional H‐type cells. Therefore, the observed selectivity trends primarily reflect differences in catalyst composition rather than mass transport limitations. These findings highlight the catalytic benefit of finely tuned Zn incorporation—typically between 5 and 30 mol%—which enhances carbon–carbon coupling and the formation of C_2_ products more effectively than undoped Cu or systems with excessive Zn content.

**Table 1:** Composition of Cu–Zn catalysts and their FE for CO_2_ reduction.

Catalyst composition	FE [%]	Products	Current density [mA cm^−2^]	Potential (vs. RHE) [V]	Cell configuration	Ref.
Zn‐doped CuO	≈45	C_2_H_4_	200	−1.0	Flow cell	[26]
Cu–Zn alloy (Cu:Zn = 95:5 mol%)	≈50	CH_3_OH, C_2_H_4_	100	−1.0	Flow cell	[33]
Catalyst Cu–Zn (Zn 20 at.%, sputtered, CV treated)	29.3	CH_3_CH_2_OH,	250	≈–0.94	Flow cell	[34]
Cu–Zn (Zn 5 at.%, sputtered, CV treated)	<29.3	CH_3_CH_2_OH, C_2_H_4_	250	≈–0.94	Flow cell	[34]
Cu–Zn (precursor Cu/ZnO,≈20 mol% Zn)	≈45	CH_3_OH, C_2_H_4_, CH_4_	20	−0.8	Flow cell	[35]
Cu–Zn (1:1 mol%)	≈30	CH_3_CH_2_OH	100	−0.8	Flow cell	[35]

These insights indicate that the role of Zn extends beyond that of a simple electronic dopant. Its capacity to modulate the electronic density of states of Cu points to a dual function: promoting carbon monoxide dimerization while simultaneously suppressing competing pathways such as the HER. This combination of effects is particularly advantageous for the rational development of efficient catalysts operating under industrially relevant conditions

### 2.2 Cu–Bi Catalysts: Enhancing Formate and Methanol Production

**Figure 2:**
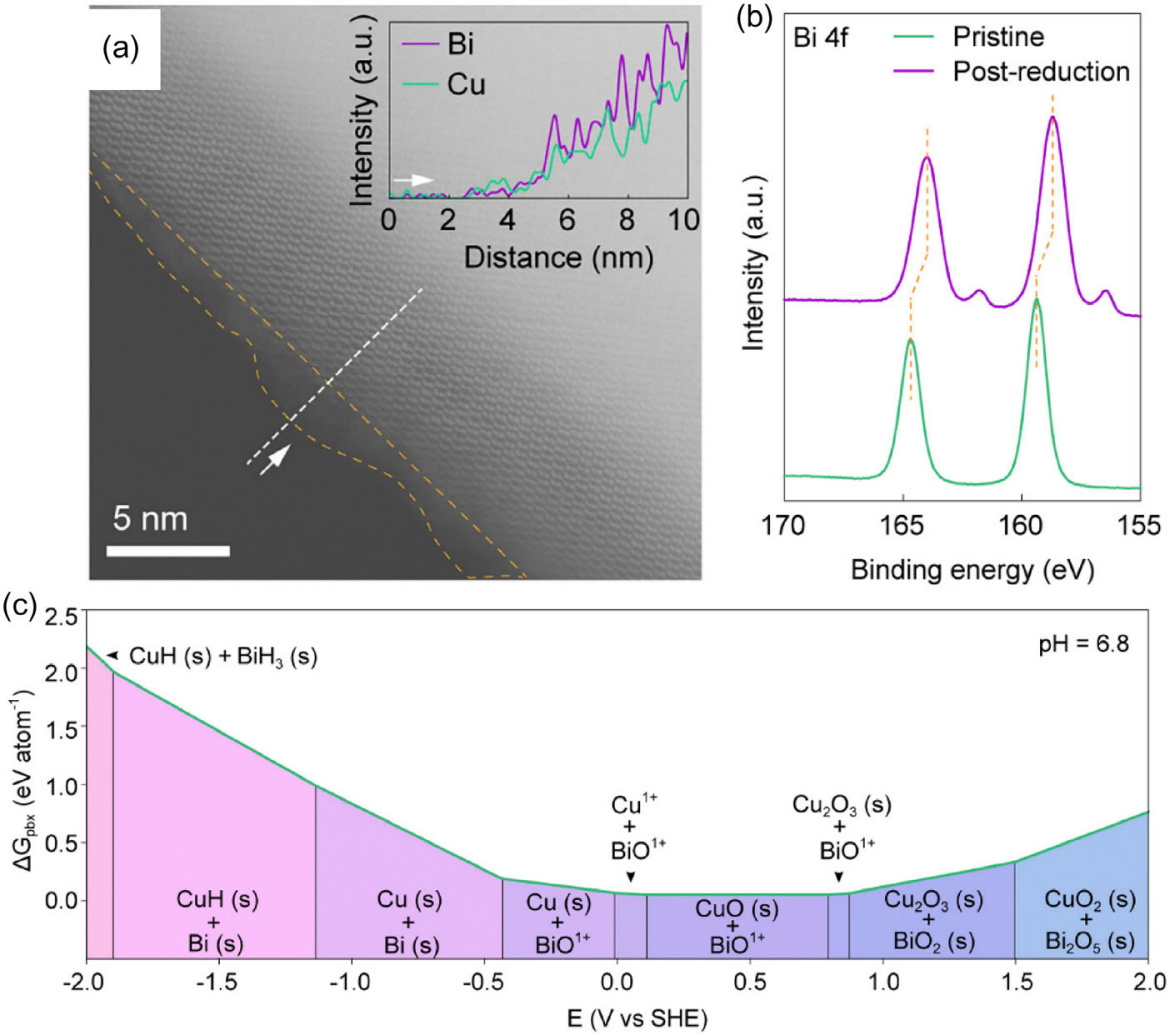
a) STEM‐ADF image of Bi segregation on the Cu surface. The yellow dashed line shows the boundary between Cu and Bi, and the white line shows the path of the EDS line scan. The inset shows the distribution of Bi on the material surface and Cu in its core. b) The XPS spectra of Bi 4f were also monitored, and the binding energies shifted upon electroreduction. c) Pourbaix diagram of the Cu–Bi system under CO_2_ reduction conditions. Reproduced from ref. [36] Published under a Creative Commons Attribution‐NonCommercial‐NoDerivatives 4.0 International License (CC BY‐NC‐ND 4.0).

Unlike Zn, Bi does not incorporate uniformly into the Cu lattice; instead, it segregates to the surface, forming an active layer that alters the material's electronic and interfacial properties.^[^ ^36^, ^37^, ^38^ ^]^ This behavior has been confirmed by scanning transmission electron microscopy–annular dark field (STEM–ADF) and energy‐dispersive X‐ray spectroscopy (EDS) line scans, which show Bi enrichment at the surface and Cu concentration in the core (**Figure** **2**a).^[^ ^36^ ^]^ X‐ray photoelectron spectroscopy (XPS) analysis of the Bi 4f region reveals that Bi is initially present as Bi^2+^, but after electroreduction, the peaks shift to lower binding energies, indicating reduction to metallic Bi (Bi^0^). This surface‐stabilized Bi^0^ phase is believed to play a key role in modifying the catalytic reactivity of the Cu–Bi interface.^[^ ^36^ ^]^

The surface segregation of Bi alters CO_2_ interactions by stabilizing oxygenated intermediates such as COOH and OCHO. Pourbaix diagrams for the Cu–Bi system indicate that CuBi_2_O_4_ decomposes into Cu and metallic Bi under negative potentials, particularly at pH 6.8 (Figure 2b,c). This thermodynamic behavior supports the experimental observations from STEM‐EDS and XPS, which confirm the accumulation of metallic Bi on the catalyst surface. Such Bi enrichment promotes the selective formation of oxygenated products like formate (HCOO^−^).^[^ ^36^ ^]^

**Figure 3:**
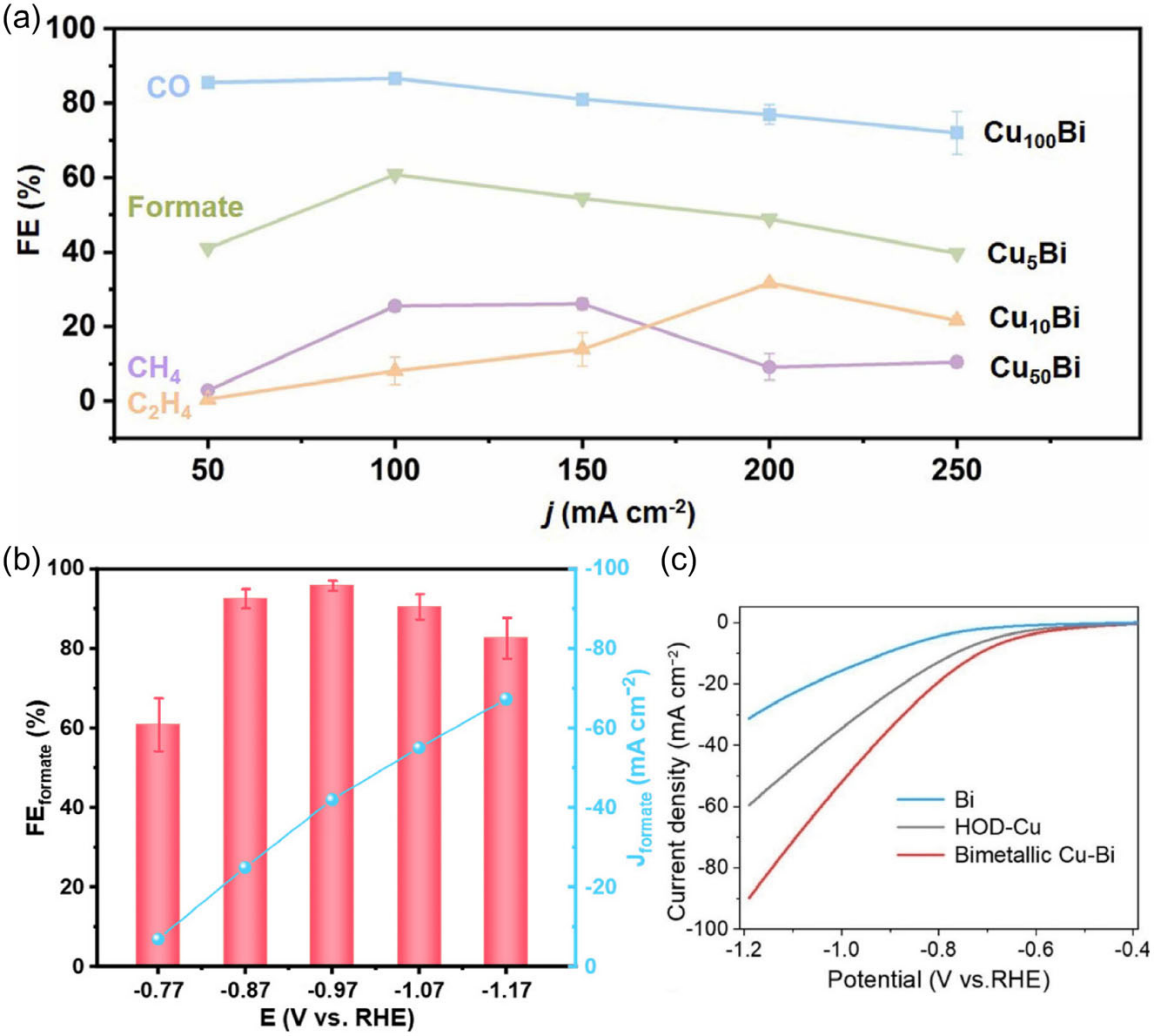
a) Faradaic CO, CH_4_, C_2_H_4_, and HCOO efficiencies at different current densities for Cu_100_Bi, Cu_50_Bi, Cu_10_Bi, and Cu_5_Bi catalysts. Adapted with permission.^[^ ^39^ ^]^ Copyright 2023, Elsevier. b) FE for formate production using Bi/OD‐Cu and Bi/Cu catalysts under CO_2_ electroreduction conditions. Adapted from ref. [40] published under a Creative Commons Attribution License (CC BY 4.0). c) Polarization curves of Cu–Bi catalysts during the electrochemical reduction of CO_2_. Adapted with permission.^[^ ^41^ ^]^ Copyright 2023, Wiley‐VCH GmbH & Co. KGaA under the STM Permissions Guidelines.

A recent study by Wang et al. (2022) demonstrated that incorporating Bi into copper enhances the production of oxygenated products during electrochemical CO_2_ reduction.^[^ ^39^ ^]^ The authors evaluated four Cu–Bi aerogel catalysts with varying Bi content. As the Bi concentration increased—particularly in the Cu_5_Bi sample—the FE for formic acid was close to 61%, with concurrent methanol formation. In contrast, the Cu_100_Bi composition, which contains no bismuth, primarily led to CO formation, while intermediate Bi loadings (Cu_10_Bi and Cu_50_Bi) favored the formation of hydrocarbons such as ethylene and methane. **Figure** **3**a illustrates the influence of Bi content on product selectivity at different current densities.

Xu et al. (2025) investigated using bismuth as a promoter for CO_2_ reduction to formate on oxide‐derived copper (OD‐Cu).^[^ ^40^ ^]^ When Bi was deposited onto a highly porous and defect‐rich copper surface, the resulting Bi/OD‐Cu catalyst achieved a FE of 97.2% for formate at–0.97 V versus RHE (Figure 3b), significantly outperforming the Bi/Cu control. This enhancement is attributed to strong electronic interactions between Cu and Bi, which improve CO_2_ adsorption and stabilize oxygenated intermediates. The study also examined how electrodeposition current density affects the growth of Bi dendrites, which in turn influences catalytic performance. The Bi/OD‐Cu catalyst demonstrated excellent long‐term stability, maintaining high efficiency throughout extended electrolysis tests. These findings underscore the importance of surface modification and substrate engineering in optimizing catalyst activity and selectivity for CO_2_ electroreduction.

Li et al. (2023) examined the use of electron‐rich Bi nanosheets deposited on Cu–Bi bimetallic structures for the electrochemical reduction of CO_2_ to formate.^[^ ^41^ ^]^ The catalyst achieved a FE of 92.5% at −1.0 V versus RHE, with a partial current density of 49.5 mA cm^−2^, as shown in Figure 3c. This high performance was attributed to electron transfer between Cu and Bi, which modified the electronic structure of Bi, enhancing CO_2_ adsorption and stabilizing oxygenated intermediates such as OCHO.

Figure 3c compares the Cu–Bi catalyst with other systems, including pure Bi and Cu nanodendrites, based on their polarization (linear sweep voltammetry) curves. The Cu–Bi system, supported on hydrothermally oxide‐derived copper (HOD‐Cu), exhibits a marked increase in current density and a more favorable onset potential, indicating superior CO_2_ conversion performance. These improvements are attributed to the high surface area and dense structural defects of HOD‐Cu, which provide an effective scaffold for Bi incorporation. The resulting bimetallic interface facilitates faster reaction kinetics and improves the adsorption and stabilization of key intermediates. A key advantage of the Cu–Bi system is its influence on the reaction pathway. Bi promotes the stabilization of the OCHO* intermediate, thereby favoring formate production over hydrocarbon formation. This modulation of the CO_2_ reduction pathway is attributed to the Cu–Bi interfacial synergy, which lowers the energy barrier for formate generation and enhances overall catalytic efficiency. Density functional theory (DFT) calculations support this conclusion, revealing that Cu–Bi catalysts create a favorable energy profile for formate formation relative to CO or hydrocarbon products.^[^ ^41^ ^]^

In Cu–Bi catalysts, the presence of Bi significantly modifies the catalyst's activity and selectivity, favoring the formation of C_1_ oxygenated products. Bi alters the interaction between Cu and CO_2_ upon surface segregation, stabilizing key intermediates such as COOH and OCHO. This effect enhances the formation of formate (HCOO^‐^) and CO, in contrast to pure Cu catalysts, which tend to favor the generation of hydrocarbons like ethylene (C_2_H_4_) and methane (CH_4_).

**Table** **2** presents the FEs for formate and methanol obtained using various Cu–Bi catalysts. The highest selectivity is achieved with 5–10 mol% Bi, reaching efficiencies above 90% for formate and over 40% for methanol under optimized conditions. These results confirm that small amounts of Bi significantly enhance oxygenate formation by stabilizing OCHO intermediates and improving CO_2_ adsorption. In addition, catalyst architecture and Cu surface properties—particularly porosity and structural defects—strongly influence selectivity. Depositing Bi onto highly porous, defect‐rich Cu surfaces reinforces the Cu–Bi interface, improves charge transfer, and promotes intermediate stabilization, enhancing product yields.

**Table 2:** Composition of Cu–Bi Catalysts and their FE for CO_2_ reduction.

Catalyst composition	FE [%]	Products	Current density [mA cm^−2^]	Potential (vs. RHE) [V]	Cell configuration	Ref.
Cu–Bi (0% Bi; Cu_100_Bi)	86.6	CO	100	–0.76	Flow cell	[39]
Cu–Bi (50% Bi; Cu_50_Bi)	26.0	CH_4_	150	–0.86	Flow cell	[39]
Cu–Bi (10% Bi; Cu_10_Bi)	31.6	C_2_H_4_	200	–0.91	Flow cell	[39]
Cu–Bi (5% Bi; Cu_5_Bi)	60.8	HCOO^−^	100	–0.91	Flow cell	[39]
Bi on oxide‐derived Cu (Bi/OD–Cu)	97.2	HCOO^−^	24	–0.97	Flow cell	[40]
Bi on metallic Cu (Bi/Cu)	92.1	HCOO^−^	40	–1.17	Flow cell	[40]
Bi nanosheets on carbon cloth (Bi NSs)	81.0	HCOO^−^	N/A	–1.0	Flow cell	[41]
Cu–Bi nanostructures on carbon cloth	92.5	HCOO^−^	49	–1.0	Flow cell	[41]
Bi–Cu catalyst electrodeposited on copper foam	94.37	HCOO^−^	28	–0.91	H‐cell	[72]
Bi_2_O_2_CO_3_@Bi nanosheets (core–shell type)	≈90	HCOO^−^	≈20	–1.07	Flow cell	[73]

These findings suggest that the role of Bi should not be regarded solely as a passive electronic modulator. Due to its ability to promote the formation of OCHO intermediates while simultaneously inhibiting C—C coupling, Bi can be strategically incorporated into tandem systems with spatially separated catalytic functions. In such configurations, Cu domains are responsible for CO_2_ activation and initial intermediate formation. In contrast, adjacent Bi‐rich regions facilitate the selective stabilization and conversion of oxygenates such as formate or methanol.

This spatial and functional segregation represents a promising strategy for tuning selectivity in CO_2_ electroreduction, particularly in applications where the formation of multicarbon products is not desired. From a catalyst design perspective, exploiting the unique properties of Bi may enable the development of highly selective systems for efficient C_1_ product generation, thereby broadening the potential of electrochemical CO_2_ valorization technologies.

## 3 Reaction Mechanisms in CO_2_ Reduction

CO_2_ reduction involves multiple interdependent steps that are strongly influenced by the structure and electronic properties of the catalyst, as well as by the nature of the intermediates formed during the process.^[^ ^42^, ^43^ ^]^ Adding Zn or Bi induces specific structural and electronic changes affecting activity and selectivity in Cu–Zn and Cu–Bi systems. In Cu–Zn catalysts, incorporation into the Cu lattice leads to lattice expansion and a downward shift in the d‐band center, which weakens CO adsorption and facilitates its desorption. This promotes the formation of C_2_ products such as ethylene and ethanol over oxygenated species.^[^ ^30^, ^32^ ^]^ Conversely, in Cu–Bi systems, Bi segregates to the Cu surface and modifies interfacial reactivity, enhancing the formation of oxygenated products like formate and methanol, while suppressing hydrocarbon production.^[^ ^36^ ^]^ These trends underscore the importance of catalyst composition and structure in governing CO_2_ electroreduction pathways.

### 3.1 CO_2_ Adsorption and Intermediate Formation

The initial step in CO_2_ electroreduction involves its adsorption onto the catalyst surface. CO_2_ is reduced via deprotonation to form CO, a key intermediate in producing multicarbon products such as ethylene on pure Cu.^[^ ^44^, ^45^ ^]^ When Zn is introduced into the Cu matrix, it facilitates CO desorption by weakening CO binding, which helps regulate surface coverage and promotes C—C coupling toward C_2_ products like ethylene.^[^ ^46^ ^]^ In contrast, Bi tends to segregate at the Cu surface and alters the CO_2_ adsorption mode, enhancing the formation of oxygenated intermediates such as COOH and OCHO.^[^ ^47^ ^]^

### 3.2 General Reaction Mechanisms in CO2 Reduction with Cu‐Based Catalysts

**Figure 4:**
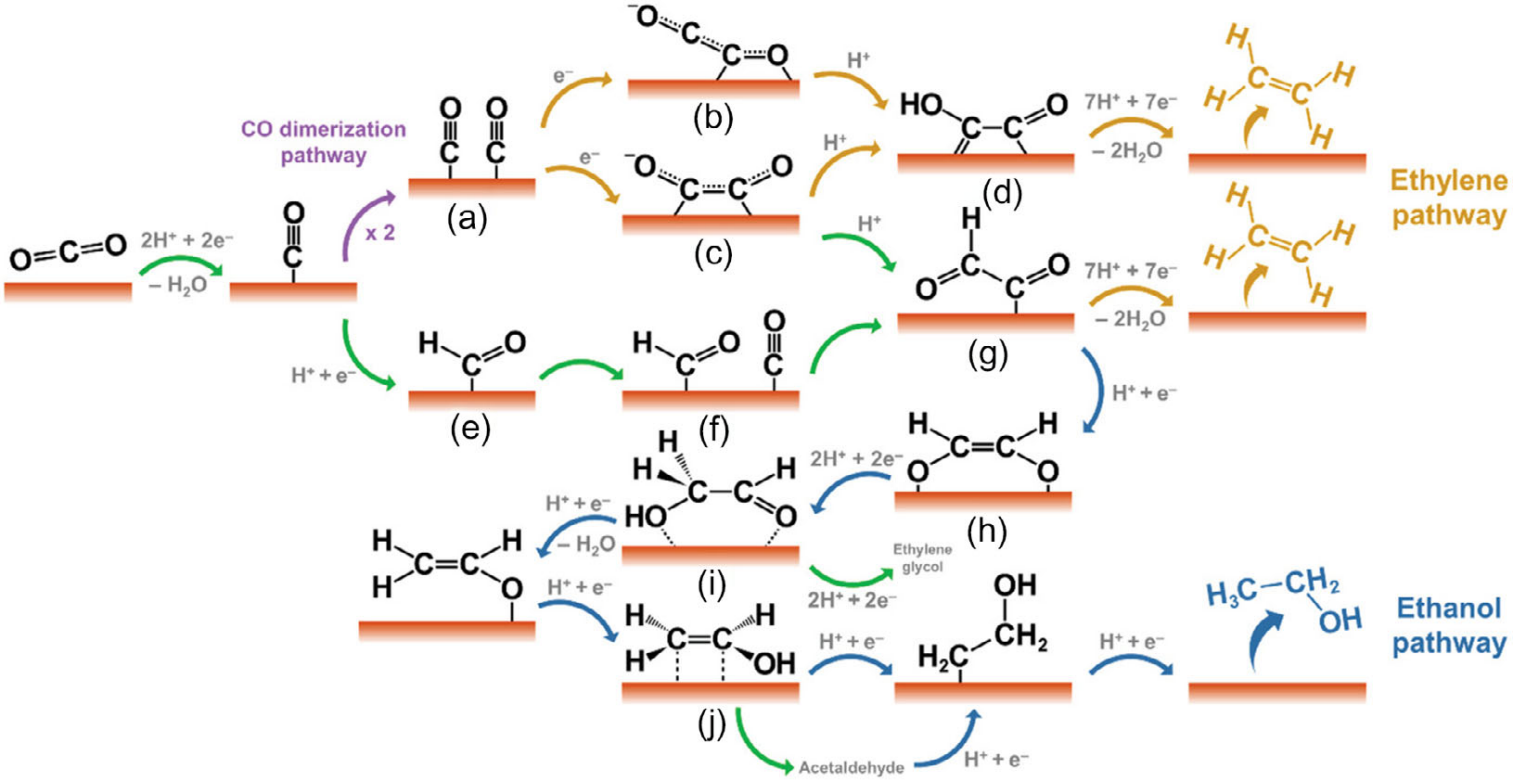
Possible CO dimerization mechanism and formation of C_2_ products in the electrochemical reduction of CO_2_ on Cu. a) *CO + *CO dimerization to form *C_2_O_2_^−^ b) CO dimerization pathway on Cu surface, leading to the formation of key intermediates. c) Direct binding of CO to the Cu surface in CO dimerization. d) Protonation of intermediates (*CO–COH and *COCHO), promoting ethylene formation. e) Formation of *CHO intermediate. f) Coupling of *CO and *CHO intermediates. g) Further reduction of *COCHO to *COCH_2_OH, leading to C_2_H_4_ formation. h) Glyoxal (C_2_H_2_O_2_) is an intermediate. i) Glycoaldehyde (C_2_H_4_O_2_) intermediate. j) Vinyl alcohol or acetaldehyde intermediates leading to ethanol formation. Adapted with permission.^[^ ^50^ ^]^ Copyright 2020, Wiley‐VCH GmbH & Co. KGaA. Reproduced with permission under the STM Permissions Guidelines.

Copper has attracted considerable attention in CO_2_ reduction research because it generates many products, including hydrocarbons such as ethane and ethylene, as well as oxygenated compounds like methanol and formate. This product diversity is primarily governed by the catalyst's crystal structure and surface composition, significantly influencing reaction pathways and selectivity.^[^ ^48^, ^49^ ^]^ A critical step in ethylene formation is the dimerization of *CO species on the Cu surface (**Figure** **4**a), forming a C_2_O_2_ intermediate. This species can adopt two configurations: in the first (Figure 4b), the intermediate binds via carbon and oxygen atoms in a bridge configuration; in the second (Figure 4c), both carbon atoms bond directly to the surface. Upon protonation, the intermediate transforms into species such as *COCHO or *CO–COH (Figure 4d), facilitating further reduction toward ethylene production.^[^ ^50^ ^]^

The authors propose that C—C bond formation occurs via coupling two *CO molecules at low overpotentials, representing a key step toward ethylene production. Subsequent reduction of *CO leads to *CHO species (Figure 4e,f). Due to the high activation energy associated with *CO dimerization, the pathway involving *COCHO becomes energetically favorable for ethylene formation. As shown in Figure 4g, *COCHO is further reduced to *COCH_2_OH, which then undergoes additional proton–electron transfers to yield C_2_H_4_.^[^ ^50^ ^]^

### 3.3 Copper Crystal Orientations and their Impact on CO_2_ Reduction

The crystallographic orientation of copper plays a vital role in the electrochemical reduction of CO_2_, as surface structure strongly influences the adsorption and desorption of key intermediates during the reaction.^[^ ^45^ ^]^ Among the most commonly studied low‐index facets are Cu(111) and Cu(100), each with distinct atomic arrangements affecting their efficiency in converting CO_2_ into methane, ethylene, and ethanol.^[^ ^51^ ^]^ In addition to these surfaces, other orientations—such as Cu(110) and high‐index planes like Cu(751), Cu(911), and Cu(322)—have also demonstrated notable catalytic activity. These high‐index facets, characterized by step edges and undercoordinated atoms, facilitate C–C coupling and enhance the formation of C_2_ and oxygenated products, thus expanding the range of catalytic possibilities beyond conventional surfaces.^[^ ^52^ ^]^

#### 3.3.1 Cu(111)‐Surface Favorable for Methane Production

The catalytic behavior of Cu(111) surfaces in the electrochemical reduction of CO_2_ has been widely studied due to their ability to produce C_1_ and C_2_ products. Zhao et al.^[^ ^53^ ^]^ applied the embedded correlated wavefunction theory to investigate reaction pathways on pristine Cu(111), showing that its highly ordered atomic structure does not stabilize *CO sufficiently for effective hydrogenation to CH_4_. Instead, this facet favors the formation of oxygenated intermediates such as *COH and *CHO, which facilitate C—C coupling and the production of C_2_ species like ethylene and ethanol. Theoretical calculations reveal that Cu(111) exhibits relatively low activation barriers (≈0.60 eV) for forming *COH–CHO adducts, enhancing selectivity toward multicarbon products.

In contrast, introducing twin boundaries into Cu(111), forming nanotwinned Cu(111), significantly alters its catalytic properties. As reported by Cai et al.^[^ ^54^ ^]^ these structural defects increase *CO adsorption strength and lower the activation energy for its hydrogenation (≈0.45 eV). This shift suppresses *CO dimerization and reduces C_2_ product formation, favoring CH_4_ generation. Therefore, while pristine Cu(111) promotes C—C coupling, nanotwinned surfaces steer the mechanism toward methanation, illustrating how atomic‐level structural engineering can be used to tune product selectivity in CO_2_ electroreduction.

Cu(111) surfaces are typically associated with high methane yields due to their strong CO binding affinity, which limits subsequent C—C coupling steps. These characteristics suggest that Cu(111)‐rich domains may be more appropriate for applications aiming at C1 product generation, where the suppression of multicarbon formation is desirable

#### 3.3.2 Cu(100)‐Surface Favorable for Ethylene Formation

Cu(100) is one of the most active copper facets for producing C_2_ products such as ethylene and ethanol during the electrochemical reduction of CO_2_. Its flat, square‐symmetric surface offers a high density of active sites that facilitate *CO adsorption and promote *CO dimerization, a key step toward C—C bond formation.^[^ ^55^, ^56^ ^]^ Recent studies have emphasized the role of *CO surface coverage in this process. Mendes et al. (2023) employed DFT calculations to investigate how varying *CO coverage influences the stability and reactivity of Cu nanoparticles.^[^ ^57^ ^]^

The results showed that as *CO coverage increases, the activation energy required for *CO dimerization to form the OCCO intermediate rises significantly—by ≈0.6 eV. Excessive *CO coverage on the surface can hinder this critical step, reducing the catalyst's efficiency in ethylene formation. However, under controlled *CO coverage, Cu(100) remains highly effective in promoting *CO dimerization. **Figure** **5** from Mendes et al. illustrates this effect: at low *CO coverage (panels A and B), the activation barrier for OCCO formation is relatively low (0.87 eV on Cu79), and the reaction proceeds via an oscillatory motion of adjacent *CO species. In contrast, at high *CO coverage (1 monolayer, panels C and D), the activation energy increases substantially—reaching values around 1.50–1.81 eV. This behavior is attributed to active site competition and modifications in the electronic structure of the surface, which hinder the adsorption and coupling of intermediates, ultimately suppressing C—C bond formation.

**Figure 5:**
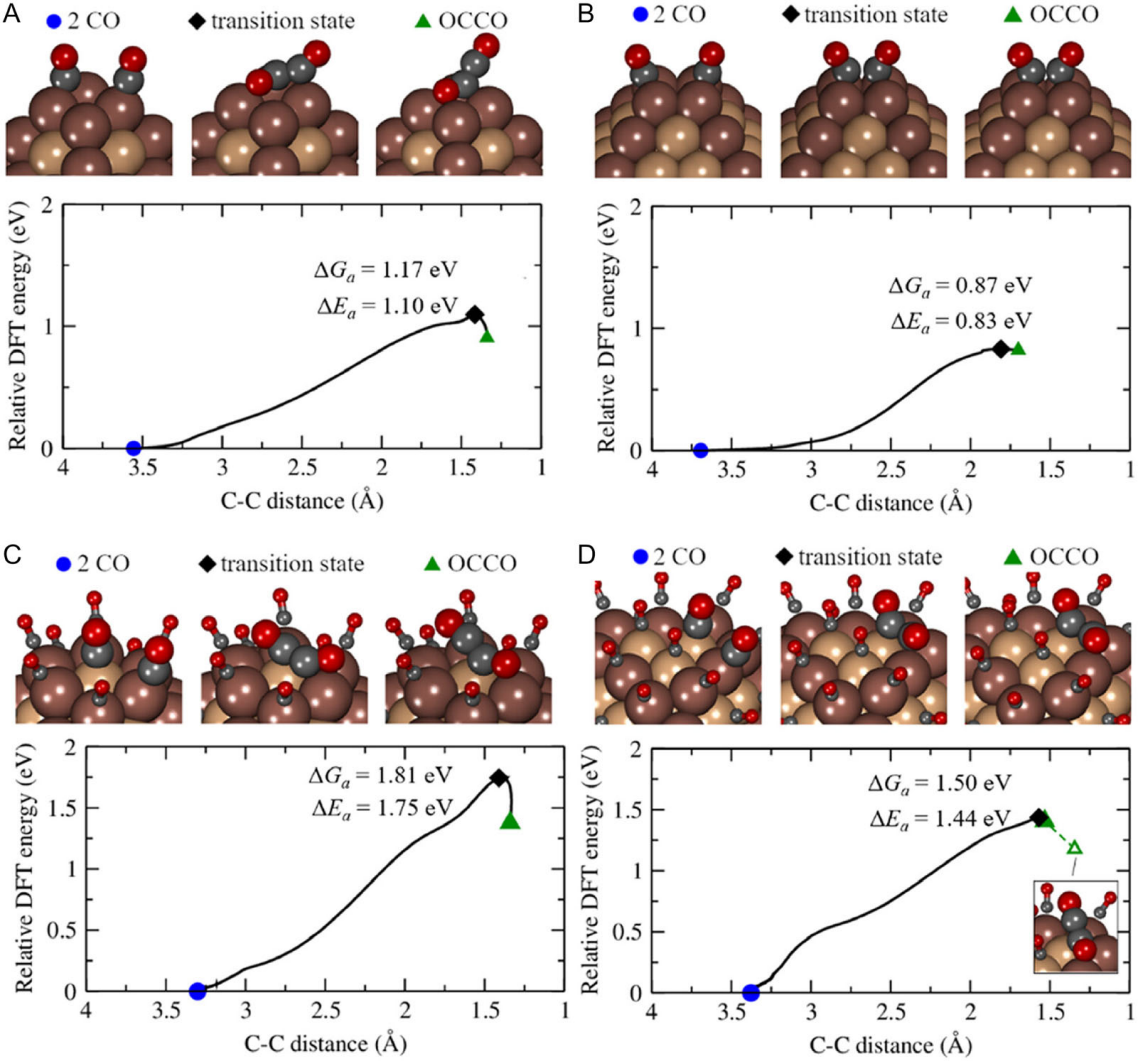
Potential energy surface for CO dimerization on Cu nanoparticles under different CO coverages. A,B) show dimerization at low CO coverage on Cu_38_ and Cu_79_, respectively, with a lower activation barrier. C,D) represent the same process at high CO coverage (1 ML) on Cu_38_ and Cu_79_, where an increase in the activation barrier is observed due to coadsorption effects. The DFT activation energies (ΔEa) and Gibbs activation energies (ΔGa) are indicated for T = 300 K. Adapted with permission.^[^ ^57^ ^]^ Copyright 2023, American Chemical Society under the STM Permissions Guidelines.

Studies by Zhao et al. (2022) and Cai et al. (2023) highlight the impact of structural modifications at Cu(100)/Cu(111) interfaces, demonstrating that even small changes in surface architecture can significantly influence the product distribution. These findings underscore that product selectivity arises not only from the presence of specific crystal orientations but also from how these surfaces interact with key intermediates such as *CO, *COOH, and *CHO. While Cu(111) surfaces generally favor methane production via *CO hydrogenation, Cu(100) enhances C—C bond formation through more favorable *CO dimerization energetics.

This comparison illustrates the importance of tailoring copper surface structures at the atomic level to maximize selectivity toward desired products. A strategic design approach that leverages the strengths of Cu(100) and Cu(111) facets can provide a rational path to enhancing CO_2_ conversion efficiency and controlling reaction pathways in electrocatalytic systems.

Surfaces enriched in Cu(100) have been reported to facilitate CO dimerization, thereby promoting the formation of C_2_ products such as ethylene. From a catalyst design standpoint, the deliberate incorporation of Cu(100)‐like domains into the material architecture emerges as a promising strategy to enhance selectivity toward multicarbon compounds under moderate overpotentials.

#### 3.3.3 Cu(110) and High‐Index Facets

Cu(110) features a more open atomic structure with lower coordination than Cu(100) or Cu(111). This configuration enhances the adsorption of key intermediates like *CO and CHO, which are crucial for C—C coupling. DFT studies suggest that Cu(110) provides favorable energetics for forming early intermediates such as COOH, thus facilitating methanol production and potentially enabling C_2_ product pathways under optimized conditions.^[^ ^52^, ^58^, ^59^ ^]^

High‐index surfaces like Cu(911), Cu(741), and Cu(322) expose a mix of terraces, steps, and undercoordinated atoms—features that improve intermediate adsorption and facilitate *CO dimerization. These surfaces offer promising energy landscapes for both coupling and protonation steps. However, their thermodynamic instability poses a challenge, as they often undergo surface reconstruction under operating conditions.^[^ ^58^, ^59^ ^]^

Despite these limitations, high‐index facets have shown great potential in model studies and nanostructures. Their structural complexity may allow for unique reactivity profiles that can be harnessed with controlled synthesis. Continued research into their stability and performance is necessary, but they offer a compelling avenue for developing next‐generation CO_2_ reduction catalysts

### 3.4 Cu–Zn and Cu–Bi Catalysts: Transition and Key Mechanisms in CO_2_ Reduction

After examining various copper crystal structures, it is essential to explore bimetallic catalysts such as Cu–Zn and Cu–Bi, which significantly enhance selectivity and activity for CO_2_ electrochemical reduction. While copper alone can convert CO_2_ into a range of products, incorporating zinc or bismuth introduces key electronic and structural modifications that guide the reaction pathway, improving efficiency and steering the process toward desired products.

#### 3.4.1 Catalyst Stability Consideration

Monometallic copper catalysts often suffer from morphological and structural instability during CO_2_ electroreduction. Under operating conditions, copper surfaces can undergo reconstruction, nanoparticle agglomeration, and oxidation–reduction cycling between Cu^0^, Cu^+1^, and Cu^+2^ species, affecting active sites’ nature and distribution and reducing catalytic performance over time.^[^ ^21^, ^60^, ^61^ ^]^

To address these issues, introducing a second metal has proven effective. In Cu–Zn systems, zinc helps mitigate copper oxidation and suppress nanoparticle aggregation, enhancing catalyst durability under cathodic conditions.^[^ ^35^ ^]^ Among different configurations, phase‐separated Cu–Zn structures have demonstrated greater resilience and improved CO selectivity than core–shell architectures.^[^ ^62^ ^]^ However, zinc is still vulnerable to leaching during extended electrolysis, which can diminish the bimetallic synergy over time.^[^ ^62^ ^]^ In contrast, Cu–Bi systems tend to exhibit higher electrochemical and morphological stability due to the inert behavior of bismuth under reductive potentials.^[^ ^63^ ^]^ Bismuth is a physical barrier that suppresses surface oxidation and copper migration, particularly when forming well‐defined interfacial regions.^[^ ^64^ ^]^ Nonetheless, the long‐term behavior of these systems remains underexplored, and further studies are required to assess their performance under continuous operation.^[^ ^65^ ^]^

Overall, bimetallic strategies like Cu–Zn and Cu–Bi improve the stability of copper‐based catalysts, but achieving robust performance under industrially relevant conditions remains an ongoing challenge.

#### 3.4.2 Cu–Zn Catalysts: Enhancing CO Production and C_*2*_ *Selectivity*

**Figure 6:**
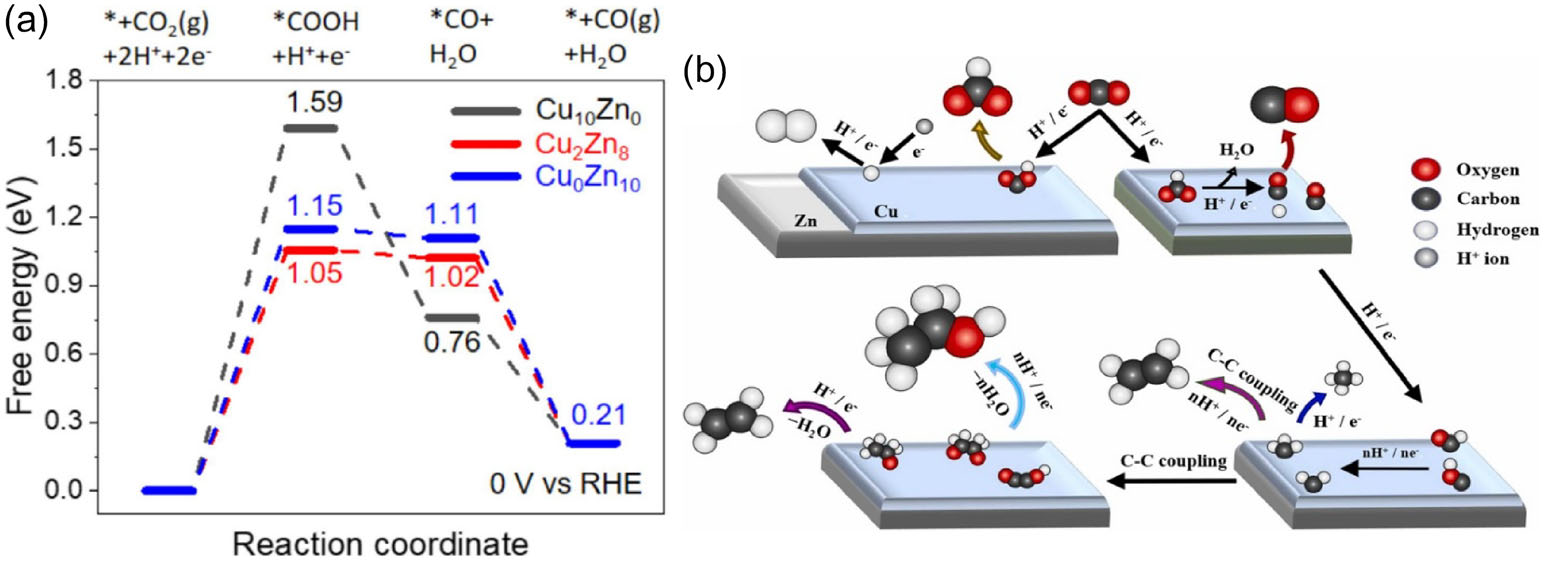
a) Gibbs free energy profiles for CO_2_ reduction to CO on Cu–Zn catalysts. Adapted with permission.^[^ ^66^ ^]^ Copyright 2023, Elsevier. b) Simplified electrochemical CO_2_ reduction mechanism based on the measured reduction products. Adapted with permission.^[^ ^67^ ^]^ Copyright 2025, Elsevier.

Properties of the active sites directly impact CO_2_ electroreduction selectivity. When Zn atoms are incorporated into the Cu lattice, they cause lattice expansion and modify the electronic density of states, thereby affecting the binding strength of key intermediates—particularly CO—and modulating reaction energetics. The Zn content plays a decisive role in determining product selectivity. High Zn concentrations promote CO formation, while lower Zn content allows the Cu surface to maintain *CO dimerization activity, leading to multicarbon products such as ethylene and ethanol. DFT calculations (**Figure** **6**a) confirm that the energy barrier for *COOH formation decreases significantly with increasing Zn content, dropping from 1.59 eV in Cu_10_Zn_0_ to 1.15 eV in Cu_0_Zn_10_ and further to 1.05 eV in Cu_2_Zn_8_. This trend aligns with the experimentally observed enhancement in CO selectivity upon Zn incorporation.^[^ ^66^ ^]^

Experimentally, Cu_2_Zn_8_ achieves a FE of 73% for CO at –1.1 V versus RHE, positioning it as one of the most efficient compositions for selective CO generation.

At lower Zn levels, the system retains catalytic features similar to pure Cu, enabling effective *CO coupling and the formation of C_2_ species. This tunability highlights the advantage of Cu–Zn alloys, where product distribution can be tailored through precise composition control and applied potential.

Figure 6b schematically represents a simplified CO_2_ reduction pathway on Cu–Zn catalysts. Zn sites favor stabilizing and desorption of *COOH intermediates, thus enhancing CO production. In contrast, Cu‐rich domains retain *CO and enable further transformation via dimerization and hydrogenation to yield C_2_ products. The interplay between these behaviors underpins the tandem nature of the system.

The Cu–Zn interface is decisive: Zn‐rich surfaces promote early CO desorption and C_1_ product formation. Cu‐rich regions retain CO, enabling C—C coupling and forming multicarbon products. This behavior is also observed in brass‐like CuZn alloys, where modified Cu–Zn interactions shift selectivity toward oxygenates such as acetate and propanol.^[^ ^67^ ^]^

**Figure 7:**
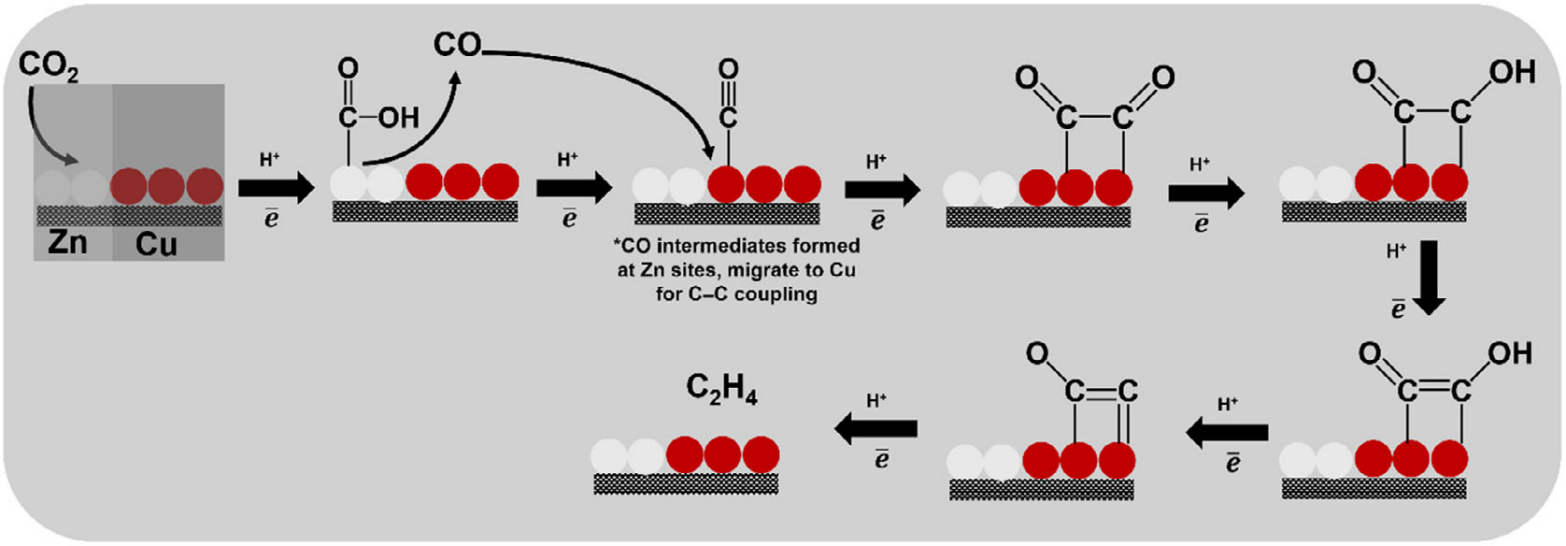
Schematic representation of the proposed tandem mechanism for CO_2_ electroreduction on Cu–Zn catalysts.

**Figure** **7** presents a conceptual model of this tandem mechanism: Zn‐rich domains catalyze the initial CO_2_ activation and *CO formation, which is subsequently utilized at adjacent Cu domains for further coupling and hydrogenation steps. This spatial separation of functions illustrates a form of cooperative catalysis, where each metal plays a complementary role—Zn as a *CO generator, and Cu as a coupling site.

The rational design of Cu–Zn architectures—where Zn domains regulate CO coverage and Cu domains facilitate C—C coupling—offers a precise approach to controlling product selectivity. Based on the presented data, combining moderate Zn content with Cu(100)‐rich surfaces under optimized conditions represents a rational design strategy to enhance CO and C_2_ product formation. Moreover, the tandem behavior intrinsic to Cu–Zn systems offers a scalable route to simultaneously access C_1_ and C_2_ products, which may be particularly advantageous for industrial implementation.

#### 3.4.3 Cu–Bi Catalysts: Enhancing Formate Production and C_1_ Selectivity

**Figure 8:**
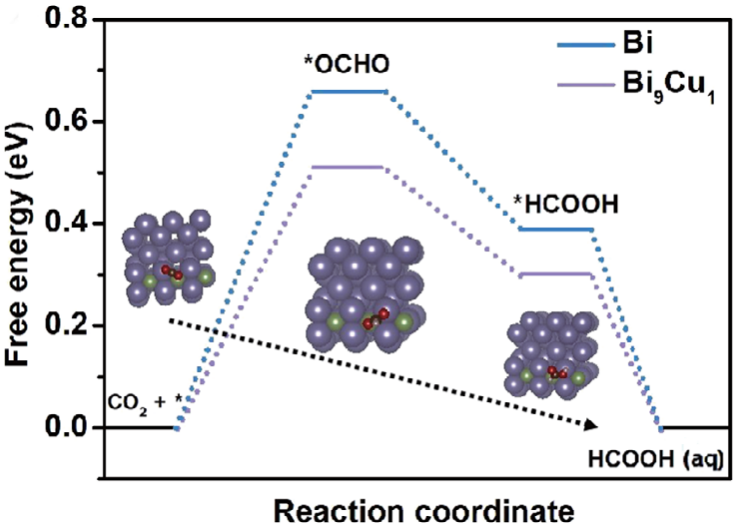
Gibbs free energy profiles showing the influence of Bi on CO dimerization and C_2_ product formation in Bi–Cu catalysts. Adapted with permission.^[^ ^70^ ^]^ Copyright 2023, Springer Nature. This content is not included under the CC BY license.

Recent studies have demonstrated that incorporating bismuth into copper‐based catalysts markedly alters their performance in CO_2_ electroreduction, particularly by enhancing the formation of C_1_ oxygenates such as formate and carbon monoxide. When Bi segregates on the Cu surface, it modulates the local electronic structure and modifies the adsorption properties of key intermediates, thereby steering the reaction toward partial reduction products.^[^ ^68^, ^69^ ^]^ DFT calculations indicate that Bi‐rich regions on Cu surfaces generate electron‐enriched sites that selectively stabilize *OCHO intermediates, critical for formate production.^[^ ^70^ ^]^ **Figure** **8** shows the calculated Gibbs free energy profiles for CO_2_ electroreduction pathways over Cu and Bi–Cu surfaces. It compares the energetics of key intermediates, revealing how the presence of Bi increases the barrier for CO dimerization while stabilizing OCHO intermediates, thus favoring formate formation over C_2_ products. This shift underscores the role of Bi in modulating *CO and *COOH adsorption energies and reactivity.

The Cu–Bi interfacial structure—mainly the Bi‐to‐Cu ratio and extent of Bi surface segregation—plays a decisive role in modulating product distribution. By tuning these parameters, the FE for formate can be maximized while simultaneously suppressing the competing HER. The strategic control of active site distribution offers a viable route to develop scalable and selective CO_2_ conversion systems.

**Figure 9:**
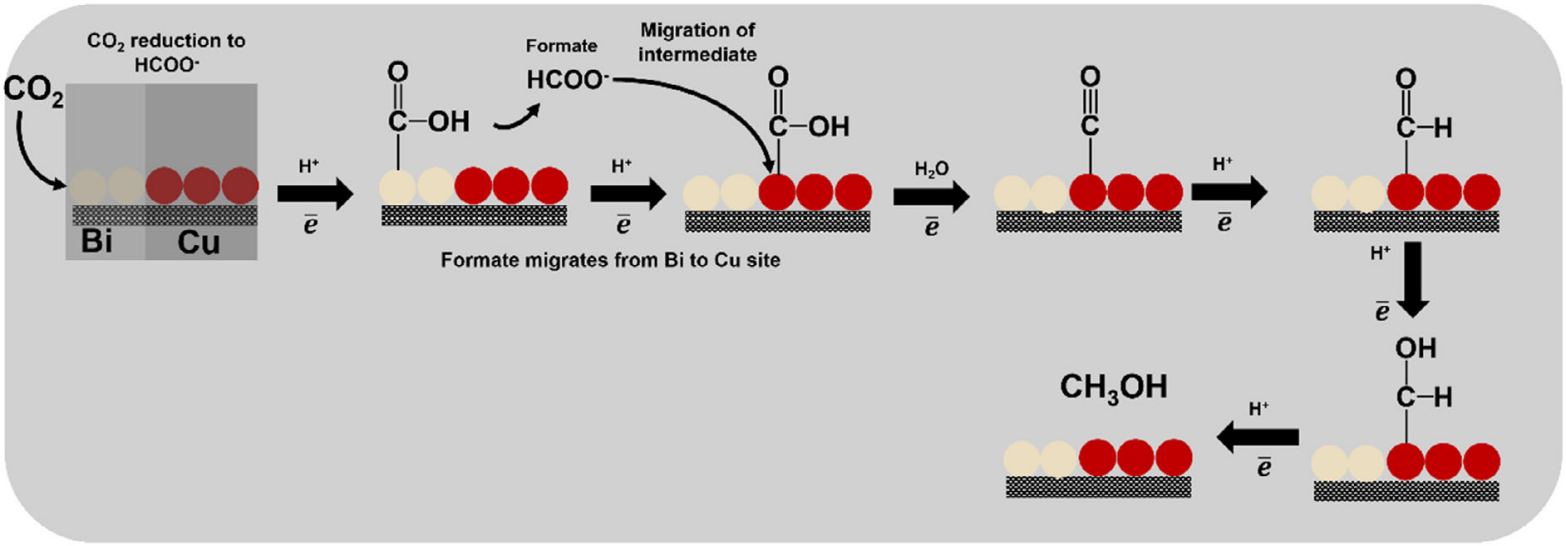
Proposed tandem mechanism for electrochemical CO_2_ reduction over Cu–Bi catalysts. CO_2_ is initially activated on Cu sites, while Bi domains stabilize *OCHO intermediates, leading to selective formate production.

A mechanistic interpretation of this tandem system is illustrated in **Figure** **9**. In this scheme, CO_2_ is first activated on Cu sites to form *CO, while adjacent Bi domains stabilize *OCHO intermediates, promoting the selective formation of formate (HCOO^−^). This spatial and functional division exemplifies tandem catalysis, where distinct active sites operate cooperatively to enhance activity and selectivity.^[^ ^71^ ^]^

This approach offers a catalyst design opportunity to modulate product distribution through selective engineering of Cu–Bi interfaces. Designing nanostructured catalysts with well‐defined Cu and Bi domains may enable precise control over intermediate stabilization, thereby advancing the development of efficient systems for C_1_ product generation under industrially relevant conditions.

## 4 Outlook and Future Challenges for Cu‐Based Bimetallic Catalysts

Despite recent progress in developing Cu‐based bimetallic catalysts for CO_2_ electroreduction, several critical challenges must still be addressed. Controlling phase segregation and achieving homogeneous dispersion of Zn or Bi within Cu matrices remains difficult, particularly under dynamic electrochemical conditions. In the case of Zn, its tendency to leach under reducing potentials poses a barrier to long‐term stability. Similarly, Bi‐based materials, while selective for formate production, often suffer from surface restructuring or deactivation during prolonged operation. Clarifying the distinct mechanistic roles of Cu, Zn, and Bi under operando conditions is also essential. While Cu is generally responsible for *CO formation and C—C coupling, Zn modifies the electronic environment and intermediate desorption, and Bi promotes *OCHO stabilization—these functions may overlap or shift depending on surface composition and applied potential. Systematic studies are still required to deconvolute these contributions and understand their interplay at the nanoscale. From a technological perspective, integrating these materials into gas‐diffusion electrodes (GDEs) or membrane electrode assemblies (MEAs) compatible with industrial current densities remains underexplored. Designs that stabilize Cu(100) facets while incorporating modulating domains of Zn or Bi—either through nanostructuring or spatial confinement—offer a promising direction for maximizing selectivity and efficiency. Ultimately, the rational design of Cu‐based bimetallic catalysts requires compositional optimization and control over surface architecture, intermediate retention, and long‐term durability. Advances in in situ spectroscopy and theory‐guided synthesis will be instrumental in addressing these challenges and moving toward scalable, high‐performance systems for CO_2_ valorization.

## 5 Conclusions and Future Perspectives

Copper‐based bimetallic catalysts remain among the most promising platforms for the electrochemical reduction of CO_2_. Cu–Zn systems promote C_2_ product formation, such as ethylene and ethanol, by stabilizing *CO intermediates and facilitating C—C coupling. In contrast, Cu–Bi catalysts exhibit outstanding selectivity toward C_1_ oxygenates like formate and methanol, driven by bismuth's electronic and structural modifications.

The catalytic performance is strongly influenced by the surface structure of copper, including the exposure of specific crystallographic facets and twin boundaries. These structural features, combined with synergistic interactions between copper and the secondary metal, govern the adsorption and stabilization of reaction intermediates and dictate product distribution.

Despite significant progress, several key challenges remain. Improving long‐term stability, mitigating metal leaching—particularly for zinc—and demonstrating high performance in industrially relevant systems such as MEAs and GDEs are critical. Notably, most studies have been conducted in H‐type cells, which do not fully replicate operational conditions in scalable technologies.

Future research should prioritize the development of robust catalyst formulations, scalable electrode architectures, and advanced operando characterization techniques capable of capturing dynamic surface transformations. A deeper mechanistic understanding and rational structure–activity design will be essential for advancing efficient, selective, and sustainable CO_2_ electroreduction technologies.

## Conflict of Interest

The authors declare no conflict of interest.

## Author Contributions

**Elías Mardones‐Herrera**: conceptualization; literature research and analysis; writing—original draft; writing—review and editing, **Mauricio Isaacs**: conceptualization; literature research and analysis; writing—review and editing; supervision, **Gonzalo García**: conceptualization; literature research and analysis; supervision, **Ilaria Gamba**: writing—review and editing. All authors have read and agreed to the published version of the manuscript.

## References

[1] US Department of Commerce, NOAA, G.M.L ., Carbon Cycle Greenhouse Gases 2023.

[2] H. Guzmán , F. Salomone , S. Bensaid , M. Castellino , N. Russo , S. Hernández , ACS ACS Appl. Mater. Interfaces 2022, 14, 517. 34965095 10.1021/acsami.1c15871 PMC8762640

[3] V. Gazzano , E. Mardones‐Herrera , N. Sáez‐Pizarro , F. Armijo , F. Martinez‐Rojas , D. Ruiz‐León , J. Honores , M. Isaacs , Front. Environ. Chem. 2024, 5, 1447014.

[4] H. Seo , T. A. Hatton , Nat. Commun. 2023, 14, 313. 36658126 10.1038/s41467-023-35866-w PMC9852473

[5] C. Castro‐Castillo , K. K. Nanda , E. Mardones‐Herrera , V. Gazzano , D. Ruiz‐León , M. J. Aguirre , G. García , F. Armijo , M. Isaacs , Mater. Chem. Phys. 2022, 278, 125650.

[6] D. Karapinar , C. E. Creissen , J. G. Rivera De La Cruz , M. W. Schreiber , M. Fontecave , ACS Energy Lett. 2021, 6, 694.

[7] A. R. Woldu , Z. Huang , P. Zhao , L. Hu , D. Astruc , Coord. Chem. Rev. 2022, 454, 214340.

[8] M. Ding , Z. Chen , C. Liu , Y. Wang , C. Li , X. Li , T. Zheng , Q. Jiang , C. Xia , Mater. Rep.: Energy 2023, 3, 100175.

[9] T. M. Gür , Prog. Energy and Combust. Sci. 2022, 89, 100965.

[10] Q. Wang , H. Wei , P. Liu , Z. Su , X.‐Q. Gong , Nano Res. Energy 2024, 3, e9120112.

[11] L. Xie , Y. Cai , Y. Jiang , M. Shen , J. C.‐H. Lam , J. Zhu , W. Zhu , Nat. Commun. 2024, 15, 10386. 39613736 10.1038/s41467-024-54590-7 PMC11607466

[12] Q. Kong , X. An , Q. Liu , L. Xie , J. Zhang , Q. Li , W. Yao , A. Yu , Y. Jiao , C. Sun , Mater. Horiz. 2023, 10, 698. 36601800 10.1039/d2mh01218a

[13] E. Mardones‐Herrera , C. Castro‐Castillo , K. K. Nanda , N. Veloso , F. Leyton , F. Martínez , N. Sáez‐Pizarro , D. Ruiz‐León , M. J. Aguirre , F. Armijo , M. Isaacs , ChemElectroChem 2022, 9, e202200259.

[14] W. Zhang , Q. Zhou , J. Qi , N. Li , React. Kinet., Mech. Catal. 2021, 134, 243.

[15] A. A. Zhang , Y. L. Li , Z. Bin Fang , L. Xie , R. Cao , Y. Liu , T. F. Liu , ACS Appl. Mater. Interfaces 2022, 14, 21050. 35476406 10.1021/acsami.2c02917

[16] G. Zhang , T. Wang , M. Zhang , L. Li , D. Cheng , S. Zhen , Y. Wang , J. Qin , Z. J. Zhao , J. Gong , Nat. Commun. 2022, 13, 1. 36522322 10.1038/s41467-022-35450-8 PMC9755525

[17] Y. F. Sun , Z. Liu , X. Liu , L. H. Gan , W. Zhang , X. Zhao , L. Bin Zhao , Appl. Surf. Sci. 2025, 681, 161469.

[18] Y. Yang , A. He , H. Li , Q. Zou , Z. Liu , C. Tao , J. Du , ACS Catal. 2022, 12, 12942.

[19] H. Li , Y. Jiang , X. Li , K. Davey , Y. Zheng , Y. Jiao , S. Z. Qiao , J. Am. Chem. Soc. 2023, 145, 14335. 37342888 10.1021/jacs.3c03022

[20] S. H. Lee , J. E. Avilés Acosta , D. Lee , D. M. Larson , H. Li , J. Chen , J. Lee , E. Erdem , D. U. Lee , S. J. Blair , A. Gallo , H. Zheng , A. C. Nielander , C. J. Tassone , T. F. Jaramillo , W. S. Drisdell , J. Am. Chem. Soc. 2025, 147, 6536. 39815387 10.1021/jacs.4c14720 PMC11869297

[21] W. Liu , P. Zhai , A. Li , B. Wei , K. Si , Y. Wei , X. Wang , G. Zhu , Q. Chen , X. Gu , R. Zhang , W. Zhou , Y. Gong , Nat. Commun. 2022, 13, 1. 35387994 10.1038/s41467-022-29428-9 PMC8986799

[22] S. Dhakar , J. Nama , V. Kumari , R. Khatua , A. Mondal , S. Sharma , Electrochim. Acta 2023, 441, 141791.

[23] M. A. H. Christiansen , A. Peña‐Torres , E. Elvar , E. Jónsson , H. Jónsson , Single Atom Substituents in Copper Surfaces May Adsorb Multiple CO Molecules, 2024. 10.1021/acs.jpclett.4c00899 38767520

[24] F. Yang , A. O. Elnabawy , R. Schimmenti , P. Song , J. Wang , Z. Peng , S. Yao , R. Deng , S. Song , Y. Lin , M. Mavrikakis , W. Xu , Nat. Commun. 2020, 11, 1. 32107389 10.1038/s41467-020-14914-9 PMC7046785

[25] T. Deng , S. Jia , S. Han , J. Zhai , J. Jiao , X. Chen , C. Xue , X. Xing , W. Xia , H. Wu , M. He , B. Han , Front. Energy 2024, 18, 80.

[26] M. H. Suliman , H. Al Naji , M. Usman , Electrochim. Acta 2024, 500, 144723.

[27] J. Zhang , C. Guo , S. Fang , X. Zhao , L. Li , H. Jiang , Z. Liu , Z. Fan , W. Xu , J. Xiao , M. Zhong , Nat. Commun. 2023, 14, 1. 36894571 10.1038/s41467-023-36926-x PMC9998885

[28] K. Wiranarongkorn , K. Eamsiri , Y. S. Chen , A. Arpornwichanop , J. CO2 Util. 2023, 71, 102477.

[29] H. Lu , N. Uddin , Z. Sun , Z. Chen , Z. Mahfoud , Y. Wu , A. A. Wibowo , Z. Su , X. Yin , C. S. Tang , X. Liao , S. P. Ringer , X. S. Zhao , H. T. Nguyen , A. T. S. Wee , M. Bosman , Z. Yin , Nano Energy 2023, 115, 108684.

[30] Y. Baek , H. Song , D. Hong , S. Wang , S. Lee , Y. C. Joo , G. Do Lee , J. Oh , J. Mater. Chem. A 2022, 10, 9393.

[31] A. Herzog , M. Rüscher , H. S. Jeon , J. Timoshenko , C. Rettenmaier , U. Hejral , E. M. Davis , F. T. Haase , D. Kordus , S. Kühl , W. Frandsen , A. Bergmann , B. Roldan Cuenya , Energy Environ. Sci. 2024, 17, 7081.

[32] I. M. Badawy , A. M. Ismail , G. E. Khedr , M. M. Taha , N. K. Allam , Sci. Rep. 2022, 12, 1. 35931804 10.1038/s41598-022-17317-6 PMC9355942

[33] Z. Wu , N. Meng , R. Yang , M. Chen , J. Pan , S. Chi , C. Wu , S. Xi , Y. Liu , Y. Ou , W. Wu , S. Han , B. Zhang , Q. H. Yang , K. Ping Loh , Angew. Chem. Int. Ed. 2025, 64, e202420283. 10.1002/anie.202420283 39653651

[34] Y. Fu , S. Wei , D. Du , J. Luo , EES Catalysis 2024, 2, 603.

[35] S. B. Varandili , D. Stoian , J. Vavra , K. Rossi , J. R. Pankhurst , Y. T. Guntern , N. López , R. Buonsanti , Chem. Sci. 2021, 12, 14484. 34880999 10.1039/d1sc04271h PMC8580038

[36] G. Zhang , B. Tan , D. H. Mok , H. Liu , B. Ni , G. Zhao , K. Ye , S. Huo , X. Miao , Z. Liang , X. Liu , L. Chen , Z. Zhang , W. Bin Cai , S. Back , K. Jiang Proc. Natl. Acad. Sci. U. S. A. 2024, 121, e2400898121. 38980900 10.1073/pnas.2400898121 PMC11260142

[37] K. Niu , L. Chen , J. Rosen , J. Björk , ACS Catal. 2024, 14, 1824.

[38] G. Jia , Y. Wang , M. Sun , H. Zhang , L. Li , Y. Shi , L. Zhang , X. Cui , T. W. B. Lo , B. Huang , J. C. Yu , J. Am. Chem. Soc. 2023, 145, 14133. 37317545 10.1021/jacs.3c04727 PMC10311520

[39] Y. Wang , L. Cheng , Y. Zhu , J. Liu , C. Xiao , R. Chen , L. Zhang , Y. Li , C. Li , Appl. Catal. B: Environ. 2022, 317, 121650.

[40] J. Xu , L. Lv , C. Wang , Y. Liang , Catalysts 2025, 15, 52.

[41] Z. Li , B. Sun , D. Xiao , Z. Wang , Y. Liu , Z. Zheng , P. Wang , Y. Dai , H. Cheng , B. Huang , Angew. Chem. Int. Ed. 2023, 62, e202217569. 10.1002/anie.202217569 36658095

[42] D. Cheng , K.‐L. C. Nguyen , V. Sumaria , Z. Wei , Z. Zhang , W. Gee , Y. Li , C. G. Morales‐Guio , M. Heyde , B. Roldan Cuenya , A. N. Alexandrova , P. Sautet , Nat. Commun. 2025, 16, 4064. 40307245 10.1038/s41467-025-59267-3 PMC12043938

[43] L. R. L. Ting , O. Piqué , S. Y. Lim , M. Tanhaei , F. Calle‐Vallejo , B. S. Yeo , ACS Catal. 2020, 10, 4059.

[44] K. Yao , J. Li , A. Ozden , H. Wang , N. Sun , P. Liu , W. Zhong , W. Zhou , J. Zhou , X. Wang , H. Liu , Y. Liu , S. Chen , Y. Hu , Z. Wang , D. Sinton , H. Liang , Nat. Commun. 2024, 15, 1. 38409130 10.1038/s41467-024-45538-y PMC10897386

[45] C. Zhan , F. Dattila , C. Rettenmaier , A. Herzog , M. Herran , T. Wagner , F. Scholten , A. Bergmann , N. López , B. Roldan Cuenya , Nat. Energy 2024, 9, 1485. 39713047 10.1038/s41560-024-01633-4 PMC11659170

[46] L. Wang , H. Peng , S. Lamaison , Z. Qi , D. M. Koshy , M. B. Stevens , D. Wakerley , J. A. Zamora Zeledón , L. A. King , L. Zhou , Y. Lai , M. Fontecave , J. Gregoire , F. Abild‐Pedersen , T. F. Jaramillo , C. Hahn , Chem. Catal. 2021, 1, 663.

[47] J. W. Shi , S. N. Sun , J. Liu , Q. Niu , L. Z. Dong , Q. Huang , J. J. Liu , R. Wang , Z. Xin , D. Zhang , J. Niu , Y. Q. Lan , ACS Catal. 2022, 12, 14436.

[48] Z. H. Zhao , D. Ren , Angew. Chem.‐Int. Ed. 2025, 64, e202415590. 10.1002/anie.202415590 39460588

[49] Q. Lei , L. Huang , J. Yin , B. Davaasuren , Y. Yuan , X. Dong , Z. P. Wu , X. Wang , K. X. Yao , X. Lu , Y. Han , Nat. Commun. 2022, 13, 1. 35982055 10.1038/s41467-022-32601-9 PMC9388520

[50] G. M. Tomboc , S. Choi , T. Kwon , Y. J. Hwang , K. Lee , Adv. Mater 2020, 32, 1908398. 10.1002/adma.201908398 32134526

[51] Y. Qiao , B. Seger , Curr. Opin. Chem. Eng. 2024, 43, 100999.

[52] K. Rossi , R. Buonsanti , Acc. Chem. Res. 2022, 55, 629. 35138797 10.1021/acs.accounts.1c00673

[53] Q. Zhao , J. M. P. Martirez , E. A. Carter , Proc. Natl. Acad. Sci. U. S. A. 2022, 119, e2202931119. 36306330 10.1073/pnas.2202931119 PMC9636923

[54] J. Cai , Q. Zhao , W. Y. Hsu , C. Choi , Y. Liu , J. M. P. Martirez , C. Chen , J. Huang , E. A. Carter , Y. Huang , J. Am. Chem. Soc. 2023, 145, 9136. 37070601 10.1021/jacs.3c00847 PMC10141442

[55] Z. Tang , E. Nishiwaki , K. E. Fritz , T. Hanrath , J. Suntivich , ACS Appl. Mater. Interfaces 2021, 13, 14050. 33705088 10.1021/acsami.0c17668

[56] H. Liu , B. Yang , Chem. Commun. 2022, 58, 709. 10.1039/d1cc06735d 34927184

[57] P. C. D. Mendes , U. Anjum , W. Lin , E. M. Smith , S. M. Kozlov , Ind. Eng. Chem. Res. 2023, 62, 20107.

[58] Q. Xue , X. Qi , K. Li , Y. Zeng , F. Xu , K. Zhang , T. Yang , X. Qi , J. Jiang , Catalysts 2023, 13, 722.

[59] G. Zhang , Z. J. Zhao , D. Cheng , H. Li , J. Yu , Q. Wang , H. Gao , J. Guo , H. Wang , G. A. Ozin , T. Wang , J. Gong , Nat. Commun. 2021, 12, 1. 34593804 10.1038/s41467-021-26053-w PMC8484611

[60] S. H. Lee , J. C. Lin , M. Farmand , A. T. Landers , J. T. Feaster , J. E. Avilés Acosta , J. W. Beeman , Y. Ye , J. Yano , A. Mehta , R. C. Davis , T. F. Jaramillo , C. Hahn , W. S. Drisdell , J. Am. Chem. Soc. 2021, 143, 588. 33382947 10.1021/jacs.0c10017

[61] I. Kim , G.‐B. Lee , S. Kim , H. D. Jung , J.‐Y. Kim , T. Lee , H. Choi , J. Jo , G. Kang , S.‐H. Oh , W. Kwon , D. Hong , H. G. Kim , Y. Lee , U. Kim , H. Kim , M. Kim , S. Back , J. Park , Y.‐C. Joo , Dae‐H. Nam , Nat. Catal. 2025, 8, 697.

[62] M. L. J. Peerlings , K. Han , A. Longo , K. H. Helfferich , M. Ghiasi , P. E. de Jongh , P. Ngene , ACS Catal. 2024, 14, 10701. 39050901 10.1021/acscatal.4c01575 PMC11264205

[63] L. Jia , H. Yang , J. Deng , J. Chen , Y. Zhou , P. Ding , L. Li , N. Han , Y. Li , Chin. J. Chem. 2019, 37, 497.

[64] S. Chang , Y. Xuan , J. Duan , K. Zhang , Appl. Catal. B: Environ. 2022, 306, 121135.

[65] Negar Sabouhanian , Jacek Lipkowski , Aicheng Chen , Ind. Chem. Mater. 2025, 3, 131.

[66] K. Zhang , W. Hua , R. Ruan , B. Cui , M. Su , X. Wang , H. Tan , Fuel 2025, 388, 134360.

[67] J. Shin , Y. Gwon , S. Y. Hwang , S. Bae , S. Y. Kim , C. K. Rhee , Y. Sohn , J. Alloys Compd. 2025, 1010, 177660.

[68] Y. Wang , W. Chen , Y. Li , J. Colloid Interface Sci. 2025, 686, 1168. 39938284 10.1016/j.jcis.2025.02.017

[69] S. Kaur , K. Garg , D. Gupta , A. Kafle , N. Dharmender , V. Shukla , R. Ahuja , T. C. Nagaiah , ACS Energy Lett. 2024, 85.38230375

[70] W. Wu , J. Zhu , Y. Tong , S. Xiang , P. Chen , Nano Res. 2024, 17, 3684.

[71] Y. Liu , F. Li , X. Zhang , X. Ji , Curr. Opin. Green Sustainable Chem. 2020, 23, 10.

[72] L. Peng , Y. Wang , Y. Wang , N. Xu , W. Lou , P. Liu , D. Cai , H. Huang , J. Qiao , Appl. Catal. B: Environ. 2021, 288, 120003.

[73] S. Liu , B. Hu , J. Zhao , W. Jiang , D. Feng , C. Zhang , W. Yao , Coatings 2022, 12, 233.

